# Putting Chagas disease on the global health agenda

**DOI:** 10.1186/s12916-023-02885-9

**Published:** 2023-05-18

**Authors:** BMC Medicine

**Affiliations:** The Campus, 4 Crinan Street, London, UK

Neglected tropical diseases (NTDs) generally most impact the poor in low-income regions. The “neglected” aspect of NTDs refers to their absence from the global health agenda resulting in limited funding from international agencies. A focused and concerted effort in improving the quality of healthcare in the most NTD-affected regions could have a huge positive impact in improving lives, yet these efforts are often hampered by a low level of awareness. Typically, when we think of NTDs dengue fever, leishmaniasis or even rabies are at the forefront of the public consciousness. This month we use our Editorial to bring awareness to Chagas disease (CD), one of the more neglected of the NTDs and the subject of April’s WHO awareness day.

CD, also currently referred to as American trypanosomiasis, is a parasitic disease caused by the protozoan *Trypanosoma cruzi*. As a vector-borne disease, the protozoa are transferred to humans in the blood meal of “kissing bugs”. Once bitten by the insect vector, individuals may proceed through an initial acute asymptomatic or minor symptomatic stage (fever, headache, lymph node and/or liver enlargement). Acute symptoms such as chronic fatigue, fever and loss of appetite are often more pronounced in children and neonates. The bite site, which is typically on the face, may also present with a swollen nodule. After the acute phase, which may last 1–2 weeks the parasite remains present, and a lifelong chronic phase begins.

A successful parasite is one which can reproduce for as long a time as possible without immediately killing the host. As such, mortality from chronic CD is usually caused by long-term effects on major organs. Chronic CD typically impacts the heart and/or gastroenterological system. Heart disease occurs in 30–40% of chronic Chagas cases. Chronic myocarditis, increased cardiomyopathy, cardiomegaly and thinning of the ventricle walls have been observed in autopsy. In 2018, *Circulation*, a journal of the American Heart Association, released an updated statement on the specifics of Chagas heart disease highlighting the need for further research on the pathogenesis of the parasite in this organ. At present, the mode of pathogenicity of the parasite in causing disease is not well understood. CD manifests in the gastrointestinal tract as an enlargement of tissues. Megaesophagus and megastomach have both been observed in chronic Chagas sufferers. Clinically, this means that individuals with chronic CD may experience arrhythmias, heart failure, sudden cardiac arrest and live with difficulty swallowing and processing foods alongside stomach pain and constipation. As such, The Institute for Health Metrics and Evaluation estimated that CD was associated with 0.275 DALYs in 2019.

Named after Dr Carlos Ribeiro Justiniano Chagas, a Brazilian physician, the disease and transmission vector was first described in Brazil in 1909. Today, CD is endemic to South and Central America with approximately 6 million individuals infected and 12000 yearly deaths. The WHO estimates that 75 million people live with the daily risk of infection. It is also estimated that 1.1 million women of childbearing age are infected with the parasite and, subsequently, 9000 neonates contract the disease via mother-to-child transmission yearly. Incidence of the disease in non-endemic regions such as Africa, Australasia and Europe is increasing. This is partly due to changing climates permitting movement of the insect vectors of the disease but largely due to migration of individuals who are unaware they are living with the disease.

Benznidazole or Nifurtimox are treatments which crucially, if utilised during the acute infection phase, are effective in complete cure of the disease. Beyond this crucial early timeframe, both the chronic infestation and subsequent effects of chronic disease are much more difficult to treat and essentially incurable. Research into therapies which can eradicate the disease in its chronic phase are ongoing but as of yet are not effective. CD is often thought of as a “silent and silenced disease” due to the long chronic phase of the disease without overt symptomology. Importantly it is estimated that of the approximately 6 million individuals living with the disease, 70% are unaware of their own infection. This gap in knowledge for the individual presents a barrier to addressing their own underlying causes of illnesses, whilst the lack of regional/countrywide data presents a major problem for public health officials in addressing this disease at the population level. The WHO’s 2023 World Chagas Day on April 14th focused on the integration of CD into primary healthcare with a specific focus on the importance of screening programs in endemic regions.

In many countries, detection rates are lower than 10% and sometimes even lower than 1%. Detection relies on serologic tests of which two are currently recommended by the WHO; a gold standard PCR test has not been established. The lack of a non-antibody-based gold-standard detection method is additionally important as CD antibodies can persist in tissues long after the parasite has been cleared from the body. As such a gold-standard diagnostic test would be essential in monitoring and assessing future therapies which may eradicate the disease in its chronic phase. Additionally, most endemic regions do not include screening for CD as part of primary care. The above issues alongside the lack of access to appropriate primary healthcare for many at risk from CD have allowed the disease to thrive in endemic regions.

Public healthcare organisations in any location are faced with the difficult task of prioritising resources. As such the issue of determining which diseases receive the most attention is literally the difference between life and death. In NTDs such as CD, the causal agents, the crucial timing of intervention required and the specific drugs with which to treat new infections are often clearly understood. In the specific case of CD, ensuring that as few people as possible proceed from acute infection into chronic infection must be prioritised by global health organisations. Comprehensive screening and treatment programs for individuals in the most impacted areas must become a focus, to ensure that the narrow window for intervention in CD is met. Without a place on the global agenda for this neglected tropical disease, the long-term consequence of this currently incurable chronic disease will continue to burden global healthcare systems.

## Data Availability

Not applicable.

